# Molecular identification and first demographic insights of sharks based on artisanal fisheries bycatch in the Pacific Coast of Colombia: implications for conservation

**DOI:** 10.7717/peerj.13478

**Published:** 2022-08-04

**Authors:** Melany Villate-Moreno, Juan Camilo Cubillos-M, Herwig Stibor, Andrew J. Crawford, Nicolas Straube

**Affiliations:** 1 Fundación MarAdentro, Bahía Solano, Colombia; 2 Aquatic Ecology, Department Biology II, Ludwig-Maximilians-Universität München, Planegg-Martinsried, Germany; 3 SNSB- Bavarian State Collection of Zoology, Munich, Germany; 4 Ecological Genomics Group, Institute of Biology and Environmental Sciences, University of Oldenburg, Oldenburg, Germany; 5 Department of Biological Sciences, Universidad de Los Andes, Bogotá, Colombia; 6 Department of Natural History, University Museum of Bergen, Bergen, Norway

**Keywords:** Marine conservation, Artisanal fishers, Population genetics, Bycatch, Eastern Tropical Pacific, Nursery area, Fisheries management, Threatened species, Genetic identification

## Abstract

The Pacific coast of Colombia is characterized by mangrove ecosystems which play a crucial role as possible nurseries for juvenile sharks. However, trophic food webs from coastal ecosystems are heavily disturbed by increased fishing pressure, which affects numerous shark species. In this region of the Eastern Tropical Pacific (ETP), fisheries’ data from coastal areas are scarce and unspecific, as most sharks from artisanal fisheries are landed decapitated and finless, making their morphological identification difficult. For the establishment and implementation of effective regional conservation and management policies, information on the diversity and population dynamics of shark species is crucial. We therefore sequenced the mitochondrial NADH2 gene of 696 samples taken from fishermen’s landings of shark’s bycatch along the Colombian north Pacific coast. We were able to identify 14 species of sharks, two of the most abundant species were *Sphyrna lewini* and *Carcharhinus falciformis*, both evaluated on IUCN the Red List of Threatened species (Critically Endangered and Vulnerable) and CITES regulated. We found low genetic diversity in the sampled area increasing the concern for both species in the region, even more considering that the majority of individuals were juveniles. Our results showed the importance of genetic markers for first population genetic insights as a complementary tool during the decision-making process in management plans. For this specific region, strategies such as the delimitation of conservation priority areas or the regulation of fishing gears could help improve the sustainability of shark populations in the Colombian Pacific.

## Introduction

Nearshore areas represent crucial environments for shark populations, offering a broad variety of habitat types and functions for different species, including foraging and mating grounds, refuge from predators, and nursery areas (Knip, Heupel & Simpfendorfer, 2010). In Colombia, the Pacific coast is characterized by estuaries, many represented by the presence of mangroves (Palacios-Peñaranda, Cantera-Kintz & Peña-Salamanca, 2019). Both of these environments play a key role as possible nurseries for juvenile sharks, because of their shallow waters, prey availability and shelter from predators (Castro, 1993; Morrissey & Gruber, 1993; Simpfendorfer & Milward, 1993; Yokota & Lessa, 2006), which are usually geographically separated from adult foraging grounds (Springer, 1967; Duncan & Holland, 2006). However, coastal regions are highly susceptible to human disturbances and exploitation, overfishing being one of the major impacts (Jackson et al., 2001) and sharks are among the most disturbed and harvested species (Nance et al., 2011).

One of the main factors contributing to shark population reduction is overfishing either targeted or incidental (Dulvy et al., 2014). The increasing demand for shark fins and meat covered by commercial fisheries, in combination with bycatch captures, have led to an alarming decline in global shark populations (Myers et al., 2007). Due to their life history strategy of slow growth rate, long life span and low reproductive rate (low numbers of progeny and late sexual maturity), shark populations have a reduced recovery potential, making them even more susceptible to overexploitation (Branstetter, 1987; Stevens et al., 2000; Clarke et al., 2015).

The Chocó department, located on the northern Pacific coast of Colombia, is one of the poorest and least studied regions of the country, despite being one of the most biodiverse areas on the planet and a global biodiversity region for conservation priority (Myers et al., 2000). In Chocó, artisanal fisheries are a valuable source of income for coastal villages, potentially forming the most important economic basis of the local economy. Catches from artisanal fisheries are either consumed locally or marketed to major cities outside the region. Even when sharks are not targeted in the area, shark bycatch is part of artisanal operations due to the use of long lines and in many cases, these catches stay unreported and species unidentified. At the moment there is a lack of scientific baseline information in the region for a better management of shark catch reduction.

Generally, a major obstacle hindering the implementation of shark conservation and management plans on a species level is the difficulty of their precise identification (Shivji et al., 2002), as well as the deficit of significant biological information on species composition (Sandoval-Castillo & Beheregaray, 2015). The collection of information related to shark catches is a main recommendation from the FAO’s International Plan of Action for the Conservation and Management of Sharks (FAO, 2000; Shivji et al., 2002). However, in Chocó, fishermen usually process their catches on their way back to shore, either for local human consumption or as bait. Captured sharks are landed unidentified, due to the removal of the fins and decapitation on board, which makes a correct morphological identification and monitoring of the captured shark species very challenging and detailed catch data such as depth or catch composition is not recorded.

The use of DNA sequence information for correct species-level identification of sharks is critical, especially when traditional taxonomic techniques are unapplicable (Holmes, Steinke & Ward, 2009). The NADH dehydrogenase subunit 2 –NADH2 is one of the fastest evolving protein-coding genes in chondrichthyans and is a well-established molecular marker for chondrichthyan species (Naylor et al., 2012a; Naylor et al., 2012b). Further, reference sequences based on voucher specimens from Naylor et al. (2012a) are available in Genbank. The mitochondrial NADH2 gene is therefore most useful to address the genetic divergence between closely related forms, cryptic species and between populations at the intra-specific level, as well as to identify unknown samples (Moore et al., 2011; Naylor et al., 2012a; Díaz-Jaimes et al., 2016), in our study a vital method for research on coastal shark diversity in the Colombian Pacific.

Since shark species differ in their specific life-history traits, their susceptibility to overexploitation also differs among species (Shivji et al., 2002). Even so, current Colombian legislation on shark fisheries lack species-specific strategies due to the gap in knowledge of population dynamics. This information is necessary for the establishment and implementation of effective regional conservation and management policies (Shivji et al., 2002). Therefore, in order to help fill in this information gap, our first goal was the examination of the shark species diversity found in artisanal fisheries bycatch on the Colombian Pacific coast based on molecular species identification. Secondly, we use the DNA sequence data to estimate genetic polymorphism, population genetic structure and connectivity within shark species in order to gain better knowledge of population dynamics on the species level. Lastly, we collect gender-size data to have an improved understanding of the demographic distribution of species in the region. The main goal of the study is gathering new genetic and first population compositional data to provide the essential scientific foundation required for the development of effective protection policies for sharks in Colombia and to establish scientific baselines for further shark research in the Eastern Tropical Pacific.

## Materials & Methods

### Study area

**Figure 1:**
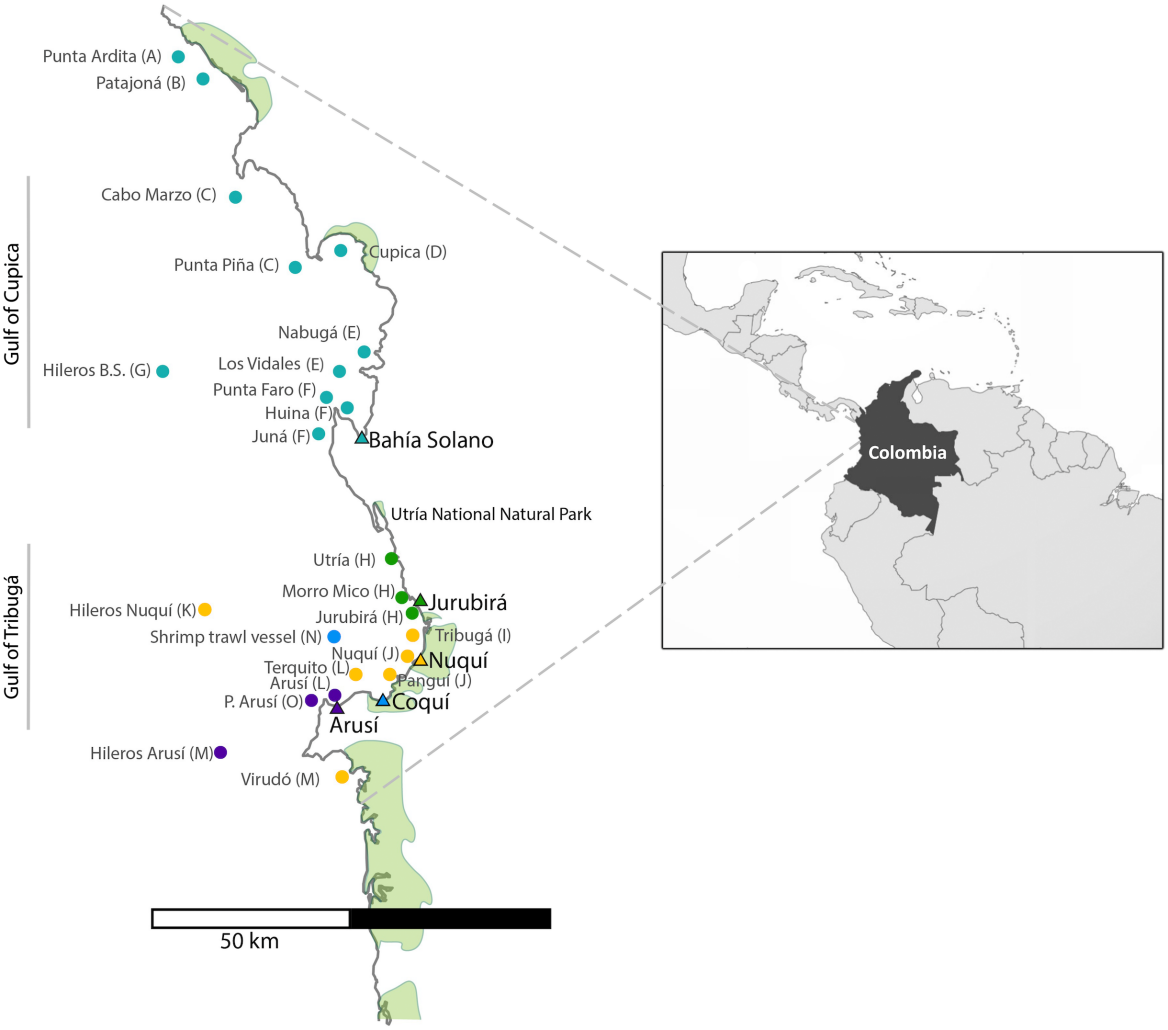
Sampling sites along the Pacific Coast of Colombia. Triangles represent the five artisanal villages where samples were collected. Each fishing site is represented by a dot which color corresponds to the village where specimens were landed. (See also Table 1). Letter codes after each locality name refer to the ‘population’ to which each fishing site was assigned for population genetic analyses.

For the collection of shark tissue samples, extensive sampling was performed along the Chocó coastline, covering a wide geographic range throughout Colombia’s northern Pacific Coast (Fig. 1). This coastal territory is characterized by rocky shores and cliffs and to a lesser degree by sandy beaches. Mangroves develop around river mouths and are mainly found in the municipalities of Juradó, Cupica, Ensenada de Utría, Tribugá, Nuquí and Coquí (Cantera, 1997; Díaz, Guillot & Velandia, 2016; López-Angarita et al., 2018; Salas, Barragán-Paladines & Chuenpagdee, 2019). The sampling area along the Pacific Coast of Colombia is divided into two zones: in the north, the Gulf of Cupica and in the south, the Gulf of Tribuga, in between both zones is the Utria National Park, where fishing is prohibited. These two geographic regions have very distinct characteristics. The continental shelf in the south extends for a much larger distance, compared to the sudden drop-off in the continental shelf at the northern end of the study area, which also influences the different species that inhabit both areas.

### Sample collection

A total of 696 muscle tissue samples of sharks were collected between 2016 and 2018, from artisanal fisheries bycatch in five coastal villages that represent the main fisheries landing sites in northern Chocó, including 466 from Bahía Solano, 141 from Jurubirá, 60 from Nuquí, 13 from Coquí and 16 from Arusí (Table 1, Fig. 1). Even though samples were collected after the landing of catches, the actual fishing sites were recorded. Samples were collected as soon as fishers landed their catches at ports or the beach. Sharks were first measured, sexed and information on capture site and the type of fishing gear used was recorded. Depending on the available time of fishers after the landing of their catches, photographic evidence was also taken. Tissue samples (approximately 250 mg) were taken directly from below the skin at areas adjacent to the dorsal or pectoral fins areas, using sterile surgical blades to avoid cross contamination. 20–25 mg of tissue was used for subsequent DNA extraction while the remaining tissue was stored at −20 °C in 96% EtOH for future analysis. Collection of samples in the field was approved by the National Authority of Enviromental Licences (ANLA) under the permit RCI [0002–00]-2021.

**Table 1:** Description of sampling sites along the Pacific Coast of Colombia. (*N* = Number of samples). The community column indicates where samples were collected and the fishing site column where shark specimens were captured. Populations are shown in Fig. 1.

Community	Fishing site	Latitude	Longitude	Population	Year	*N*
Bahía Solano	Punta Ardita	7.128096	−77.814408	A	2016–2018	51
	Patajoná	7.101236	−77.785007	B	2016–2018	5
	Cabo Marzo	6.824408	−77.672435	C	2016–2018	64
	Punta Piña	6.635790	−77.537177	C	2016–2018	71
	Cupica	6.670012	−77.462803	D	2016–2018	45
	Nabugá	6.403539	−77.370571	E	2016–2018	56
	Los Vidales	6.367989	−77.450043	E	2016–2018	28
	Juna	6.177708	−77.485994	F	2016–2018	48
	Huina	6.293682	−77.431619	F	2016–2018	18
	Punta Faro	6.306900	−77.472684	F	2016–2018	18
	Hileros Bahía	6.382700	−78.107956	G	2016–2018	40
	Unknown	–	–	–	2016–2018	22
Jurubirá	MorroMico	5.871837	−77.312561	H	2017–2018	17
	Jurubirá	5.817369	−77.288947	H	2017–2018	83
	Utría	5.917953	−77.346459	H	2017–2018	41
Nuquí	Tribugá	5.792841	−77.268268	I	2017–2018	22
	Nuquí	5.714915	−77.282344	J	2017–2018	14
	Pangui	5.671582	−77.315431	J	2017–2018	1
	Hileros Nuquí	5.824215	−78.088735	K	2017–2018	21
	Terquito	5.641564	−77.417673	L	2017–2018	1
	Virudó	5.423225	−77.440346	M	2017–2018	1
Coquí	Shrimp trawl vessel	5.938292	−77.781123	N	2017–2018	13
Arusí	Arusí	5.609987	−77.485343	L	2017–2018	6
	Punta Arusí	5.607141	−77.484426	O	2017–2018	6
	Hileros Arusí	5.466847	−78.078441	M	2017–2018	4

### DNA extraction, PCR amplification and DNA sequencing

Genomic DNA was extracted from muscle tissue using the DNeasy Blood and Tissue kit (QIAGEN Inc, Germany); DNA extracts were stored at −20 °C in elution buffer until further processing. Shark specimens were identified by amplifying the mitochondrial NADH dehydrogenase subunit 2 gene (NADH2). Polymerase chain reaction (PCR) amplification was performed using primers ILEM (5′- AAG GAC CAC TTT GAT AGA GT -3′) and ASNM (5′- AAC GCT TAG CTG TTA ATT AA -3′) (Naylor et al., 2012b). Each PCR reaction contained 18.2 µL of ddH2O, 2.5 µL of 10X RED PCR buffer (Steinbrenner^®^), 2 µL dNTPs (Steinbrenner^®^), 1 µL each of forward and reverse primers, 0.2 µL of SB-Taq Polymerase (Steinbrenner^®^) and 0.8 µL of DNA template, for a total volume of 25.7 µL. The PCR cycling conditions comprised an initial denaturing step of 15 min at 95 °C, followed by 35 cycles at 94 °C for 30 s, 55 °C for 30 s, 72 °C for 1 min and a final 10 min extension at 72 °C. PCR amplification success was checked by gel electrophoresis and samples with low or no amplification were re-tested using 1.5 µL of DNA template. PCR products were purified by EtOH precipitation. Each cycle-sequencing reaction contained 4.05 µL of ddH2O, 0.50 µL of SB buffer (Steinbrenner^®^), 0.15 µL each of forward and reverse primers, 0.30 µL of BigDye terminator v3.1 (Applied Biosystems^®^) and 1 µL of DNA template, for a total volume of 6 µL. Posterior dye terminator removal was performed with magnetic MagSi-DT Removal Beads (Steinbrenner^®^) and then sequenced in both directions at the LMU Sequencing Service in Munich using BigDye terminator v3.1 kit (Applied Biosystems^®^) on an Applied Biosystems^®^ Sanger sequencing instrument (ABI 3730 capillary sequencer).

Forward and reverse sequences were edited and contigs assembled using Geneious 7.1.9 (http://www.geneious.com, Kearse et al., 2012). Consensus sequence alignments were inferred, and each DNA sequence was translated into amino acids to check for stop codons. The resulting alignment contained 1044 aligned nucleotides and no inferred gaps.

### Molecular species identification

Species identification was performed in two steps: first, we used the Basic Local Alignment Search Tool (BLAST) (Altschul et al., 1990), to compare sequences generated in this study to the public database, GenBank at NCBI (National Center for Biotechnology Information, http://www.ncbi.nlm.nih.gov/genbank/). We considered a species ID reliable if the similarity percentage was >98% and the Expect value (E) smaller than e−4. Once the species were identified, available NADH2 sequences for these species were downloaded from GenBank and aligned with our sequences, to infer a molecular phylogeny of our shark samples. *Hypanus longus* was used as an outgroup. The placement and divergence patterns of our newly obtained sequences relative to the published data was checked using a clustering approach with a neighbor-joining (NJ) analysis in MEGA 11 (Tamura, Stecher & Kumar, 2021), assuming the Tamura-Nei substitution model, identified as the best-fitting model by MEGA based on our generated sequences. We used 10,000 bootstrap replicates for evaluating statistical support for each node of the NJ tree (Felsenstein, 1985).

### Species distribution—abundance, maturity stages and sex

Most shark specimens were finned and beheaded by fishermen before landing and typically only the Trunk Length (TrL) could be obtained. In only a few instances (*N* = 38) was it possible to measure the Total standard Length (TL). We further measured the Interdorsal Space Length (IDS), *i.e.,* the distance between the posterior end of the anterior first dorsal fin and the anterior end of the second dorsal fin (*N* = 696). All measurements were repeated measuring in the same order. Sex was recorded whenever pelvic fins where still attached.

For size class estimation, a morphometric analysis was performed using the length at maturity for four of the 14 species for which size at maturity information was available. To estimate specimens’ TL, IDS measurements were used following the equation TL = *β* + (α*IL) (Polo-Silva et al., 2017; Santana-Hernandez, Tovar-Avila & Valdez-Flores, 2014). Where *β* and *α* represent specific values for each species based on reported morphometric relationships from populations in the Eastern Pacific (Pérez-Jiménez, Rocha-Olivares & Sosa-Nishizaki, 2013; Polo-Silva et al., 2017).

### Genetic diversity and population structure

We estimated genetic polymorphism and population structure for all species in our dataset for which we obtained at least 20 samples, in order to maximize precision. NADH2 diversity was first visualized using median-joining (MJ) network of haplotypes in PopART (Leigh & Bryant, 2015) independently for each species.

Population genetics summary statistics per species and fishing site were calculated in Arlequin 3.5 (Excoffier & Lischer, 2010), including haplotype frequency, number of polymorphic sites (S), number of transitions and transversions and nucleotide composition. For detecting genetic diversity the number of NADH2 haplotypes, haplotype diversity (h) and nucleotide diversity (*π*) were estimated with Nei’s corrected average genetic divergence ( Nei, 1987) and assuming Tamura & Nei’s (1993) model of sequence evolution.

The extent of genetic differentiation throughout the sampling range based on NADH2 sequences was assessed using an analysis of molecular variance—AMOVA implemented in Arlequin 3.5 (Excoffier & Lischer, 2010), using the Tamura-Nei model of sequence evolution. Fishing sites were grouped together into “populations” (as shown in Table 1) according to their geographic closeness and different hypotheses were tested to evaluate population structure. The first hypothesis considered each population as a separate unit, testing genetic differentiation among populations. For the second hypothesis, populations A, B, C, D, E, F and G located on the Gulf of Cupica, were grouped together. Populations H, I, J, K, L, M, N and O located on the Gulf of Tribuga, were also grouped together, in order to test if there was a higher genetic differentiation between groups than within. This group distinction was based on the differences in bathymetry and management of the marine territory between the north and south Gulfs.

Phi-ST (an mtDNA analog of Wright’s *F*-statistics; Wright, 1965) were calculated between populations. Statistical significance of pairwise population comparisons was assessed by comparison to a null distribution of 10,000 samples generated by permuting haplotypes between populations. The initial 0.05 significance level was adjusted for multiple comparisons using the Bonferroni approach (Rice, 1989).

## Results

A total of 689 shark specimens were successfully sequenced (98.9% of the samples) for the mitochondrial NADH2 gene from five sampling villages, comprising 24 fishing sites along the Pacific Coast of Colombia. All aligned sequences had a length of 1,044 bp (348 amino acids).

### Molecular species identification

In total, we identified 14 shark species belonging to three different families: Carcharhinidae (five species), Sphyrnidae (five species) and Triakidae (four species). The family Triakidae was the most abundant family in terms of the number of individuals caught, with a total of 349 sharks, followed by Sphyrnidae with 223 individuals and Carcharhinidae with 117. *Mustelus lunulatus* was the most abundant species (33%), followed by *Sphyrna lewini* (30%) (Table 2).

**Table 2:** Molecular species identification. Relative frequency and IUCN conservation status of each identified species, based on mitochondrial NADH2 gene sequence data of sharks landed from bycatch along the northern Pacific Coast of Colombia (*N* = Number of individuals).

*N*	Frequency	Family	Species	IUCN Status
228	33%	Triakidae	*Mustelus lunulatus*	Least Concern
212	30%	Sphyrnidae	*Sphyrna lewini*	Critically Endangered
108	15%	Triakidae	*Mustelus henlei*	Least Concern
72	11%	Carcharhinidae	*Carcharhinus falciformis*	Vulnerable
24	3.4%	Carcharhinidae	*Carcharhinus limbatus*	Vulnerable
13	1.9%	Carcharhinidae	*Rhizoprionodon longurio*	Vulnerable
8	1.15%	Triakidae	*Mustelus albipinnis*	Least Concern
7	<1%	Carcharhinidae	*Carcharhinus cerdale*	Critically Endangered
5	<1%	Sphyrnidae	*Sphyrna zygaena*	Vulnerable
5	<1%	Triakidae	*Mustelus* sp.1	Not available
3	<1%	Sphyrnidae	*Sphyrna media*	Critically Endangered
2	<1%	Sphyrnidae	*Sphyrna tiburo*	Endangered
1	<1%	Sphyrnidae	*Sphyrna corona*	Critically Endangered
1	<1%	Carcharhinidae	*Carcharhinus leucas*	Vulnerable

**Figure 2:**
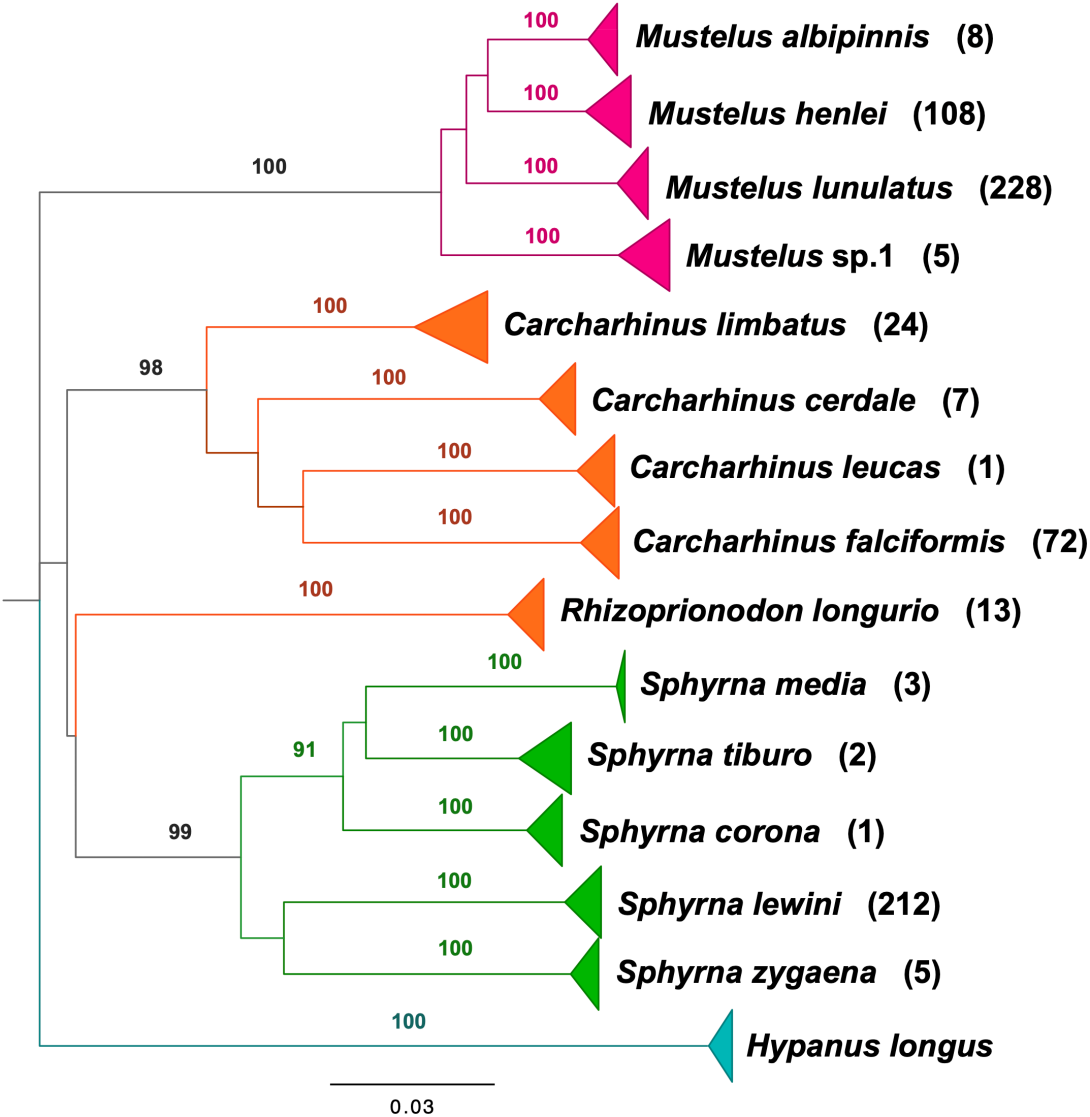
Neighbor-joining tree based on mitochondrial NADH2 DNA sequence data. Shark species landed from artisanal fisheries bycatch at the Pacific coast of Colombia and reference sequences from Naylor et al. (2012a) and Naylor et al. (2012b). Tree was rooted using *Hypanus longus* as the outgroup. Numbers above each branch are bootstrap values based on 10,000 replicates, and only values >95% are shown. Numbers in parentheses are the amount of analyzed sequences per species. Colors represent the four taxonomic families. Magenta = Triakidae, Orange = Carcharhinidae, Green = Sphyrnidae, turquoise = Dasyatidae.

BLAST analyses showed that all shark samples matched reference sequences from Naylor et al. (2012a) and Naylor et al. (2012b) with an identity percentage of >98%. The neighbor-joining tree of all samples represents the phylogenetic relations among shark samples collected in the Colombian Pacific, supporting 14 clusters, 13 of which correspond to a described species, with high bootstrap support (Fig. 2). For Triakidae, three monophyletic species-specific clades were obtained: *Mustelus lunulatus*, *Mustelus henlei, Mustelus albipinnis* as well as a clade of a potentially undescribed species labeled *Mustelus* sp. 1. The five different clades belonging to the Sphyrnidae family contained *Sphyrna lewini, Sphyrna zygaena, Sphyrna media, Sphyrna tiburo* and *Sphyrna corona*. Similar results were obtained for the Carcharhinidae, with five species-specific clades for *Carcharhinus falciformis, Carcharhinus limbatus, Rhizoprionodon longurio, Carcharhinus cerdale* and *Carcharhinus leucas*.

**Figure 3:**
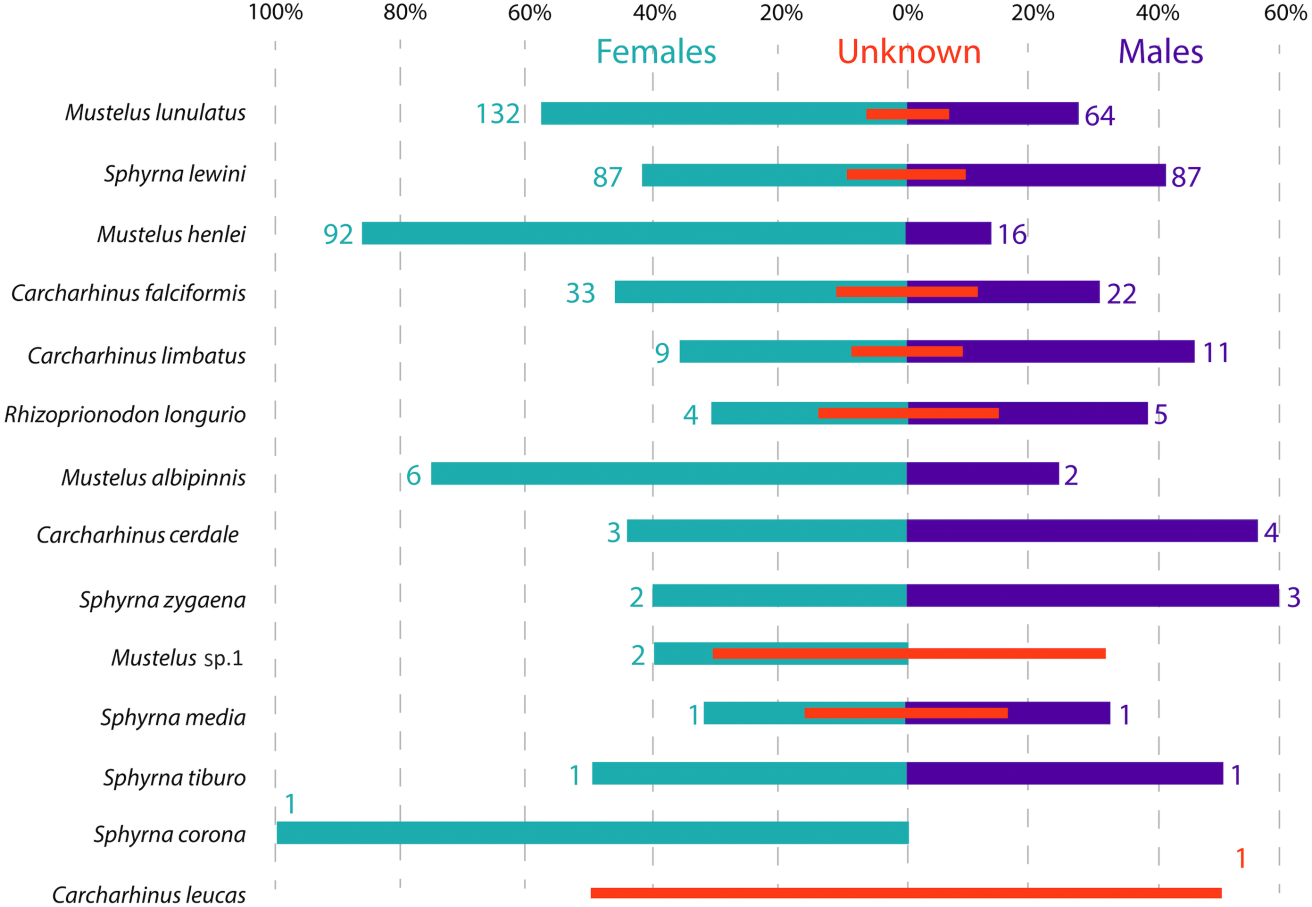
Sex ratio of each shark species landed from artisanal fisheries bycatch along the Pacific Coast of Colombia, assessed with morphological data. Numbers flanking bars represent the absolute number of individuals per species per sex, with red bars indicating the % of samples in each category that could not be sexed. For categories with no samples that could be sexed, the absolute number of individuals is also indicated by a red number.

### Species distribution—abundance, maturity stages and sex

None of the species reported here were found in all fishing sites. *M. lunulatus* and *S. lewini* were the most widespread, both found in 10 of 15 fishing sites. *Sphyrna lewini* was most commonly found in the Jurubirá region (Fig. 1). Nabuga, one of the bays in the northern part of Choco, was the place where four of the five *Sphyrna* species in the Colombian Pacific waters were found, all of which were recorded in this study. *Carcharhinus falciformis* was mainly found in deep waters off the continental shelf (Hileros BS and Hileros Nuqui) (Fig. 1); a few individuals of smaller size were found closer to the coast. *Carcharhinus limbatus* was found in fishing sites located close to mangrove areas and was not a commonly captured species. *Mustelus henlei* and *M. albippinis* were exclusively found in the northern part of the study area and *M. henlei* was also found at a shrimp trawling boat location, a deep-water locality. *Mustelus* sp. 1*.* was only found in the Cabo Marzo –Piña area and it was captured during the three sampling years in that same fishing site.

Across all samples, the overall sex ratio was biased towards females (1.72:1), with 376 females, 220 males and 101 unsexed individuals in total across all species (Fig. 3). In the case of *M. lunulatus*, despite males being present at all fishing sites, the ratio was 2:1 in favor of females, and they were most abundant in Cabo Marzo. In *M. henlei* the ratio of females to males was 5.7:1 and males were only found in three of the nine sites where the species was captured. One of these sites was the shrimp trawling boat location, where the proportion of females and males was equal and no individuals of *M. lunulatus* were found. For almost all species of the Sphyrnidae and Carcharhinidae the ratio of females:males was 1:1 except for *C. falciformis,* where it was 1.5:1 towards females.

**Figure 4:**
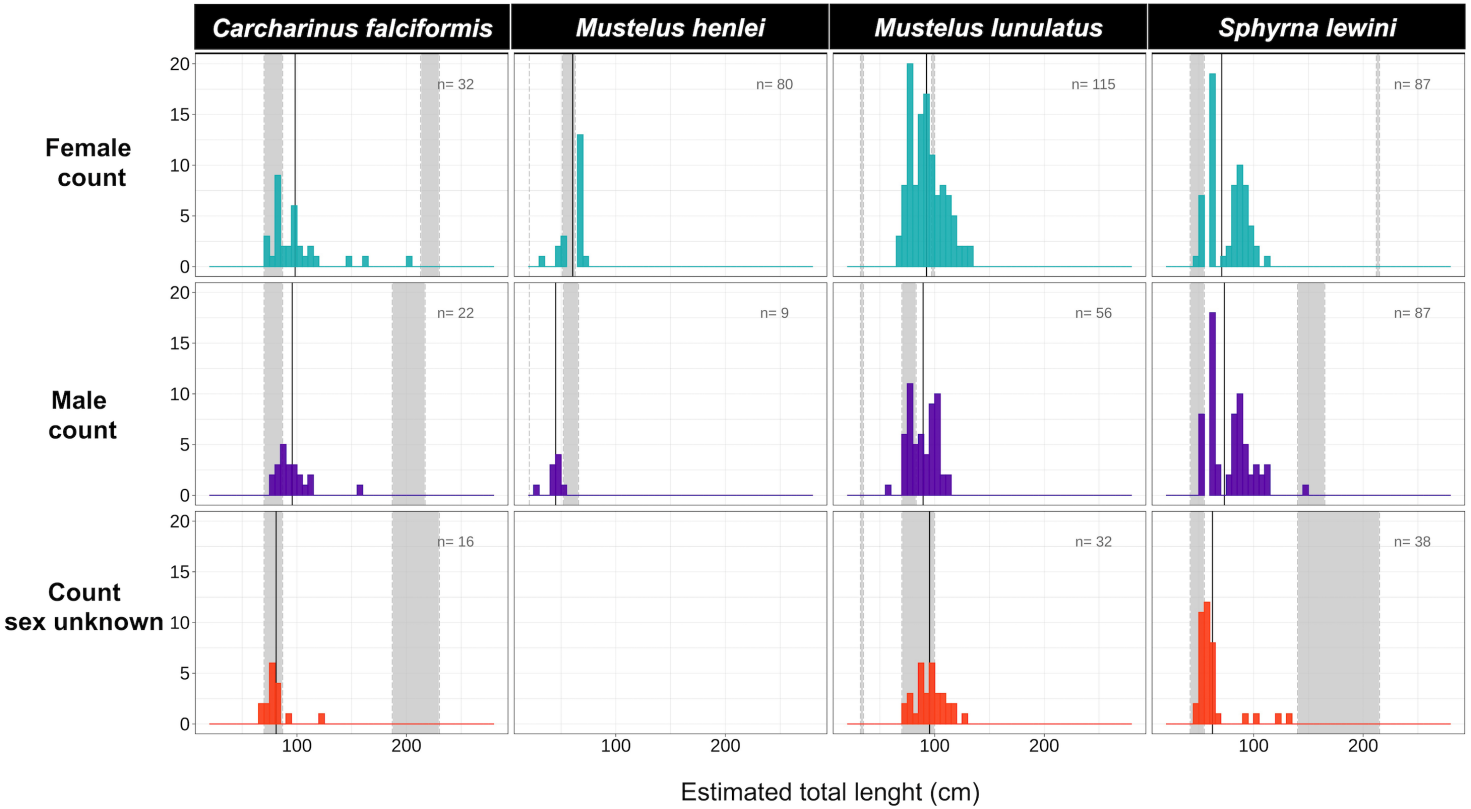
Size-class estimation (juveniles or adults) after morphometric analyses were performed for each shark species, using species-specific information on length at maturity. Measures are separated by species and sex. For each histogram, the grey bar on the right represents the range of sizes at maturity based on Pérez-Jiménez et al. (2013) and Polo-Silva et al. (2017); the grey bar on the left represents the neonate size range for the species. Shown here are four of the 14 species for which size at maturity information was available.

Body-length estimates from the interdorsal space (IDS) length indicated that all individuals of *C. falciformis* and almost all (with one exception) of *S. lewini* were juveniles. For both species, individuals within the neonate size range were found as well; the minimum and maximum size per species are shown in Fig. 4. In contrast, for *M. lunulatus* none of the individuals were within the neonate size range and 70% were below the minimum maturity size. For *M. henlei,* 16% of sampled individuals were below the minimum maturity size and none were neonates (Fig. 4**)**. Overall, 19 specimens of *S. lewini* were measured for TL and IDS allowing for the estimation of a linear regression and for comparison with those calculations previously described by Polo-Silva et al. (2017).

### Genetic diversity and population structure

**Figure 5:**
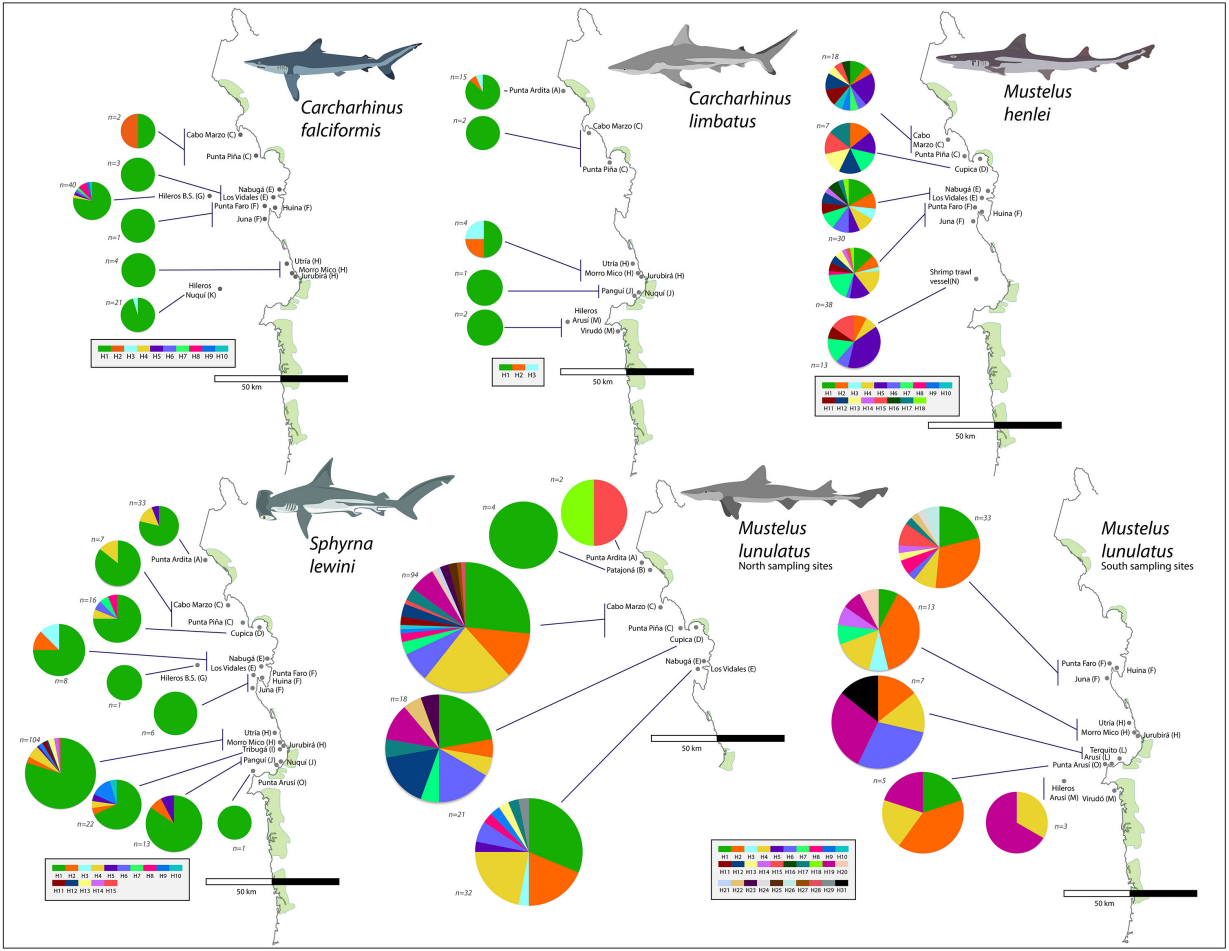
Haplotype frequencies obtained from the mitochondrial NADH2 gene sequence from 644 specimens corresponding to the five most frequently caught shark species obtained from artisanal bycatch. The map is showing fishing sites in the Pacific Coast of Colombia. Only the fishing sites where the respective species was fished are shown in the map. For *Mustelus lunulatus*, haplotypes distribution is divided in two maps for better assessment of the results.

From the 696 sampled individuals, 644 were included in the population genetic analysis. A total of 77 haplotypes characterized by 78 polymorphic sites were found among the five species for which we obtained >20 DNA sequences (Table 3). The levels of haplotype and per-site nucleotide diversity were greatest in *M. henlei* (*h* = 0.917, *π* = 0.00241) and *M. lunulatus* (*h* = 0.868, *π* = 0.00254) and lowest diversity in *C. falciformis* (*h* = 0.283, *π* = 0.00029) (Table 3). The abundance and frequency of each haplotype as well as the nucleotide composition per species are found in Table S1 and Table S2. No specific fishing site or area held the highest genetic diversity for all studied species at once. On the contrary, each species presented a unique pattern of genetic diversity distribution as observed in Fig. 5.

**Table 3:** Population genetic summary statistics for the mitochondrial NADH2. For the five shark species that had N > 20 samples: number of haplotypes (NH), number of polymorphic (segregating) sites (S), haplotype diversity (h) and nucleotide diversity (*π*) estimated with Neis corrected average genetic divergence (Nei 1987).

Species	*N*	*N*H	*S*	*h*	*π*
*Carcharhinus falciformis*	72	10	9	0.283	0.00029
*Carcharhinus limbatus*	24	3	2	0.304	0.00031
*Mustelus henlei*	108	18	20	0.917	0.00241
*Mustelus lunulatus*	228	31	31	0.868	0.00254
*Sphyrna lewini*	212	15	16	0.367	0.00065

*Carcharhinus falciformis*: The 72 sequences resolved 10 haplotypes defined by nine polymorphic sites, eight of these haplotypes were singleton haplotypes, meaning a unique, unshared haplotype, only present once in the samples (Luzier & Wilson, 2004). Six of these singletons were found at the same site (G) (Fig. 5). Hap one, the most common haplotype, was shared by 61 individuals and found in all seven populations. The highest genetic diversity in this species was found in region G, which also corresponds to the fishing site where most of the samples were obtained (*h* = 0.4).

*Carcharhinus limbatus*: In this species, two polymorphisms resulted in three haplotypes. There were no singletons, and the most common haplotype was shared by 20 individuals and found in all five populations (Fig. 5). The highest diversity in this species was found in region H (Fig. 1- Table 1) (*h* = 0.833).

*Mustelus henlei:* There were 20 polymorphic sites, resolving 18 haplotypes; of these, three were singletons. Hap five was the most common one, found in 17 individuals and widespread across all six populations where this species was recorded (Fig. 5). Haplotypes two and seven were also found in all six populations, but in fewer individuals. This species showed high genetic diversity at all fishing sites, the highest one found in region D (Fig. 1- Table 1) (*h* = 1.0).

*Mustelus lunulatus*: The 228 sequences resolved 31 total haplotypes defined by 31 polymorphic sites, 11 of these haplotypes were singletons. The most common haplotype (Hap 1) was present in 58 individuals; meanwhile Hap 4 was the most widespread found in eight of the 10 populations (Fig. 5). The greatest diversity was found in region D (Fig. 1- Table 1) (*h* = 0.91).

*Sphyrna lewini:* There were 16 polymorphic sites resolving 15 haplotypes and seven singletons were found. The most common haplotype was Hap 1, shared by 168 from 212 individuals and present in all 10 populations (Fig. 5). Overall, this species showed low genetic diversity at all fishing sites, the highest diversity found in region I (Fig. 1- Table 1)(*h* = 0.532).

For all species except *M. lunulatus,* AMOVA results indicated the lack of significant genetic heterogeneity (*p* > 0.05) between the northern and southern groups for the NADH2 at the Pacific Coast of Colombia. The largest proportion of genetic variability for every species was found within populations, rather than among populations within the two groups or between groups. In the case of *M. lunulatus*, a small but significant Φ _CT_ value of 0.017 (*P*-value = 0.04) was obtained, indicating some degree of heterogeneity among the northern and southern groups, however, after the Bonferroni correction, the differentiation was not significant. Following this same pattern, only pairwise comparisons among *M. lunulatus* populations showed significant genetic differentiation (*P* < 0.05) (Table 4).

**Table 4:** Estimated pairwise Φst values of *Mustelus lunulatus* for 10 populations (below diagonal) and corresponding *p*-values (above diagonal), based on mitochondrial DNA NADH2 sequences.

	C	D	F	E	B	A	L	M	H	O
C	———	–	–	–	0.015^*^	–	–	–	–	–
D	0.01451	———	–	–	–	–	–	–	0.024^*^	–
F	−0.01055	0.02926	———	–	0.021^*^	–	–	–	–	–
E	−0.00793	0.00013	−0.01496	———	0.041^*^	–	–	–	–	–
B	0.27694	0.11925	0.30913	0.24123	———	–	0.009^*^	0.002^*^	0.001^**^	–
A	0.11269	0.18649	0.06921	0.02649	0.82268	———	–	–	–	–
L	0.00774	0.07239	−0.01819	−0.00880	0.56931	0.00130	———	–	–	–
M	−0.08925	−0.11306	−0.06490	−0.11798	0.57952	0.16002	−0.06025	———	–	–
H	0.03698	0.14015	0.01217	0.02972	0.58956	0.04494	−0.01093	0.09867	———	–
O	−0.09073	−0.05240	−0.09553	−0.10553	0.50048	0.07610	−0.07499	0.20056	0.05791	———

**Notes.**

Population abbreviations are explained in Table 1. Only significant *p*-values are shown.

* *p* < 0.05.

** *p* < 0.001, after Bonferroni correction.

## Discussion

Our study is the first genetic identification and detailed examination of shark species population composition including demographic insights, diversity, abundance, and population structure in the Pacific Coast of Colombia based on artisanal fisheries bycatch. From the identified species, ten (71%) have been assessed as either Critically Endangered (four), Endangered (one) or Vulnerable (five) by the IUCN Red List of threatened species (https://www.iucnredlist.org) (Table 2). This study also provides the first record of the species *M. albipinnis* in Colombian waters. Additionally, we report on a mitochondrial sequence cluster, which cannot be assigned to any described species so far, indicating cryptic diversity within the genus *Mustelus* in the Colombian Pacific (Fig. 2). *Mustelus* sp. 1 was already discussed in Naylor et al. (2012a) to be an additional and independent cluster likely representing an undescribed species. We found haplotypes of the same genetic cluster in our analysis. In an ongoing project, we are securing voucher specimens of this cluster for additional morphological analysis.

In this study, 100% of samples for *C. falciformis*, 99.5% of *S. lewini* and 70% of *M. lunulatus* were juveniles found in the area over the 2016–2018 sampling period, suggesting that the northern Chocó mangroves and estuaries of Colombia are acting as nursery grounds.

However, defining a shark nursery area cannot only be based on the occurrence of juvenile sharks; species abundance, residency and inter-annual use of the area also need to be considered (Heupel, Carlson & Simpfendorfer, 2007). Although some of this information is still lacking, the dominance and abundance of juveniles and their habitat use over time can be considered a strong indicator that the Northern Chocó area could be used communally as a nursery by at least three shark species. Communal use of a nursery area by different shark species could be advantageous to reduce adult predation on juveniles (Simpfendorfer & Milward, 1993). Neonates and juveniles tend to spend the initial part of their life within the nursery, where there is higher access and abundance of prey (Simpfendorfer & Milward, 1993; Duncan & Holland, 2006; Springer, 1967), factors that help to counterbalance mortality rates in young sharks (Heupel & Simpfendorfer, 2002; Yokota & Lessa, 2006) and therefore increase recruitment into the adult population (Knip, Heupel & Simpfendorfer, 2010).

Since a representative and statistically relevant number of individuals was not obtained for every species reported in this study, population genetic analysis and conclusions are based on the five most frequently captured species that represent 93.4% of all sampled specimens. At the top of the list were two species of *Mustelus*, the sicklefin smooth-hound (*M. lunulatus*) and the brown smooth-hound (*M. henlei*). Both are coastal water, demersal mesopredatory sharks, nevertheless they inhabit different depths and showed a different distribution in this study. *Mustelus lunulatus* was found in almost every fishing site, except at the deeper ones far away from shore (the species is generally found in areas between 0–100 m deep (Ferrera-Rodríguez, 2014)). *M ustelus henlei* was only found at the northern part of the sampling area and at the shrimp trawling location. The reason may be the bathymetric differences between northern and southern sampling a, *i.e.,* a wider shelf falling less steep into the abyssal plain in the north. Both species showed the highest genetic diversity in the area, which could be related to their intrinsic life-history characteristics. They present similar life history traits such as relatively high fecundity, early maturity, and annual reproductive cycles. *Mustelus lunulatus* size at maturity is approximately 97–103.2 cm for females and 70–91.5 cm for males, having six to 19 pups per litter (Compagno, 1984): (Pérez-Jiménez & Sosa-Nishizaki, 2010; Méndez-Macías et al., 2019). *Mustelus henlei* size at maturity is approximately 51–63.5 cm for females and 52–66 cm, for males, having 1–10 or 1–21 pups per litter depending on their geographic location (Yudin, 1987; Yudin & Cailliet, 1990; Pérez-Jiménez & Sosa-Nishizaki, 2008; Chabot & Haggin, 2014; Soto-López et al., 2018).

In fisheries, the resilience of a stock is related to the reproductive characteristics of a species (Soto-López et al., 2018). Sharks such as *M. lunulatus* and *M. henlei* have rather high fecundity and short reproductive cycles and thus relatively high productivity among elasmobranchs (Walker, 2005; Soto-López et al., 2018), which may help the species secure a higher recruitment rate relative to mortality by fishing pressure (Feitosa et al., 2018). This, coupled with the fact that both species mature at an early age, increases the probability that captured individuals have already reached sexual maturity and produced offspring (Fig. 4). Therefore, both species are likely able to tolerate higher rates of fishing pressure in comparison to other elasmobranch species.

The presence of only females of *M. henlei* at most sites (Fig. 3) could be explained by sex-specific segregation, where females and males inhabit different depths or have different latitudinal distribution frequencies, which has been shown for several other triakid species and have previously been described as a possible strategy related to reducing mating encounter rates. (Chabot & Haggin, 2014). Here, at two of the three sites where males of *M. henlei* were present, we observed a 1:1 ratio between females and males, possibly representing a mating location for this species in the area. It would be important to know if this pattern of female-only sites and “mating locations” occurs throughout the year or seasonally. In the case of *M. lunulatus*, Navia, Giraldo & Mejía-Falla (2006) reported that for the Pacific Coast of Colombia, *M. lunulatus* individuals larger than 50 cm do not present spatial sexual segregation. Our data supports these results, since both females and males were found in the majority of sites where the species was captured. However, the sex ratio in most of these sites was 2:1 in favor of females. The higher ratio of females to males in both of these species also supports the idea of the usage of this shallow coastal environment for pupping and subsequent nursing.

The Scalloped Hammerhead (*S. lewini*) was the second most commonly captured shark documented here. They are heavily fished worldwide either targeted or as bycatch and highly prized and traded in the global fin trade (Villate-Moreno et al., 2021; Pinhal et al., 2020). As a result, severe population declines have occurred in different regions of the world and therefore *S. lewini* was evaluated as Critically Endangered in the most recent (Rigby et al., 2019) assessment and is listed under the Appendix II of CITES. It is a large bodied, highly migratory species with a coastal-pelagic life history and global distribution in tropical and temperate waters (Chapman, Pinhal & Shivji, 2009; Daly-Engel et al., 2012; Hadi et al., 2020; Pinhal et al., 2020).

Spatial distribution of this species displays complex patterns of segregation, with juveniles, adult females and adult males occupying different habitats (Klimley, 1987). Scalloped hammerheads make use of near-shore areas as nursery grounds (Duncan & Holland, 2006). This was evidenced in our study, where juveniles were found to inhabit shallow areas close to the coast. Females display site fidelity to coastlines, returning to shallow coastal areas to reproduce and give birth to pups, which stay seasonally residing in this area for a few years (Chapman, Pinhal & Shivji, 2009; Daly-Engel et al., 2012). The degree of this fidelity to specific nursery areas is still under debate and our results indicate that for the Colombian Pacific, the degree of genetic differentiation among possible nursery areas is not significant. Therefore, females possibly stray between proximal nursery areas, as has been previously observed (Duncan et al., 2006; Daly-Engel et al., 2012).

Similar to many other shark species, *S. lewini* is k-selected; combined with high fishing pressure these factors make them highly vulnerable to overexploitation (Branstetter, 1987; White, Barton & Potter, 2008). Even though they give birth to 2–30 pups per litter, this amount might not be high enough for successful population recovery (Branstetter, 1987). Females mature at approximately 240–250 cm and males mature at approximately 180–200 cm in length (Branstetter, 1987; Hazin, Fisher & Broadhurst, 2001). At least a decade is necessary for offspring to reproduce and contribute to the maintenance of the population, since females mature at approximately 15, and males around 9–10 years of age (Branstetter, 1987; Hazin, Fisher & Broadhurst, 2001).

Our sampling consisted exclusively of juvenile specimens harvested by fisheries, which clearly reflects that management and protective measures considering nursery areas are not in place at the moment and should be established in the area. Given the current critically endangered status of this species, results from studies like ours, based on genetic diversity, population dynamics and first demographic insights are much needed as a foundation for the development of sustainable measures for the maintenance of shark populations in the ETP. The degree of fisheries exploitation, coupled with low genetic diversity at mitochondrial DNA (Table 3; Fig. 5) and reliance on estuarine nursery grounds, makes these sharks extremely susceptible. It is of special concern that in the Pacific Coast of Colombia the population exhibiting the highest degree of genetic variability was the Tribuga fishing site, located next to Jurubirá, where the species was most frequently found. This area is currently under consideration as a significantly large harbor site, increasing the degree of concern for this species in the Colombian Pacific. Furthermore, considerably low genetic diversity was detected in *S. lewini*, even lower when compared to other studies for this species in nearby areas of the Eastern Pacific (Duncan et al., 2006; Nance et al., 2011). A possible explanation for such low genetic variability could be the age of these populations in the Colombian Pacific. According to Duncan et al. (2006), the Eastern Pacific was recently colonized by a dispersal event (∼100 000 years ago) from the oldest extant population of *S. lewini* in the Indo-west Pacific using the archipelagos of the South Pacific and Hawaii as stepping-stones for colonization across the Pacific. In general, younger populations tend to have lower genetic diversity, as they are generally founded by few individuals and have less time for mutation to fixate and generate more diversity, in comparison to older populations.

Among the five species analyzed herein, *C. falciformis* showed the lowest genetic diversity in the area (*h* = 0.276), only 10 haplotypes in 74 sampled individuals were found (Table 3). Accordingly, Galván-Tirad et al. (2013) identified only nine haplotypes for the mitochondrial control region (mtCR) among 242 sampled individuals in the Eastern Pacific Ocean, demonstrating relatively low genetic variation as detected in *S. lewini*. However, Clarke et al. (2015) who also sequenced the mtCR in silky sharks, reported on finding high genetic diversity on a global scale (*h* = 0.86), as well as one of the highest nucleotide diversities in comparison to other shark species. Therefore, the lack of genetic diversity of this species in the Colombian Tropical Pacific region is probably not a signature of a historical lack of diversity in the species*,* but possibly due to a founder effect phenomenon, where the Eastern Pacific Ocean population is the result of the Western Pacific Ocean ancestral population expansion by few members (Galván-Tirad et al., 2013), as has been shown in other shark species (Keeney & Heist, 2006; Duncan et al., 2006). Another reason for low genetic variation could be the result of more recent population reductions by overexploitation, especially since relative abundance of silky sharks in the Eastern Pacific Ocean has decreased approximately by 90% (Galván-Tirad et al., 2013).

Silky sharks are globally distributed in tropical and subtropical regions, reaching a maximum size of 330–350 cm (Compagno, 1984; Bonfil, 2008; Sánchez-de Ita et al., 2010). They are considered as a moderately (Bonfil, 2008) to fast growing species (Branstetter, 1990), with females maturing at approximately 210–225 cm and males at approximately 202–250 cm (Branstetter, 1987; Bonfil, 2008; Sánchez-de Ita et al., 2010), however for the Eastern Pacific Ocean, a smaller size at maturity (180 cm) has been reported (Cadena-Cárdenas, 2001). Juveniles and adults inhabit different areas; newborns and juveniles have been reported to reside in deep waters on the continental shelf, while adults prefer pelagic habitats, residing in deeper waters off the continental shelf or oceanic waters (Bonfil, 2008; Sánchez-de Ita et al., 2010; Clarke et al., 2015). This is consistent with our results where the majority of individuals (juveniles) were found at the “Hileros” sites, which are fishing locations far from the shore (approx. 50–60 miles). It is precisely due to their pelagic habits and distribution that silky sharks are directly affected by target or bycatch fisheries in open waters and their populations have severely declined during the last decades, contributing as well in great proportion to the global fin trade (Clarke et al., 2015; Sánchez-de Ita et al., 2010; Villate-Moreno et al., 2021). In addition, here we report artisanal fisheries catching juveniles in coastal areas, which increases the fishing pressure on this species in the ETP.

The blacktip shark (*C. limbatus*) is a medium-sized species (approximately 2 m in length) and inhabits the tropical and subtropical waters around the world (Castro, 1996). Our results suggest that the blacktip shark is not a common bycatch species in the Colombian Pacific. Females reach sexual maturity at around 117–132 cm and males at approximately 103–110 cm (Carlson, Sulikowski & Baremore, 2006). The species is considered to have a moderately slow growth rate and low fecundity, having only four to six pups after an 11–12 month gestation period (Castro, 1996; Keeney et al., 2003; Keeney et al., 2005). They are currently classified as Vulnerable by the IUCN, their populations are susceptible to stock collapse (Keeney et al., 2003; Keeney et al., 2005) since their life history traits limit the species recruitment capacity and blacktip sharks constitute a significant component of shark fisheries in other areas of the ETP.

Currently, there are no genetic studies of blacktip sharks including individuals from the Eastern Pacific Ocean on the South American coastline. Among the 24 individuals characterized here, three haplotypes were recovered which differ only in a single nucleotide suggesting a very low haplotype and nucleotide diversity in the region. In contrast, previous genetic studies based on the mtCR marker showed much higher genetic diversity for this shark species both in the US Atlantic and Gulf of Mexico (*h* = 0.710, *π* = 0.00106, Keeney et al., 2003) including the Caribbean Sea (*h* = 0.805 *π* = 0.00214, Keeney et al., 2005). The low genetic diversity found in this study could be attributed to the sample size, however, Keeney et al. (2003) suggested that the low levels of genetic diversity found in some nursery areas from the Atlantic coast could be related to a significant reduction of the effective female population size in the area due to a former bottleneck event. This could also be the case for blacktip shark populations in the Colombian Tropical Pacific where the abundance of the species may be considerably lower compared to populations from other areas of the world.

This reduced genetic variation is consistent with Bowen et al.’s (1998) ‘olive ridley turtle’s regional extirpation hypothesis’, as a consequence of significant temperature reduction in the Eastern Pacific. Bowen et al. (1998) proposed that after this local extirpation, re-colonization of the Eastern Pacific region took place by turtle populations from the Indo-Pacific Ocean during the last 300,000 years. Accordingly, Keeney & Heist (2006) suggested the same hypothesis could be appropriate for blacktip shark populations from the Eastern Pacific. The authors found haplotypes being shared among the Philippines, Hawaii, and the Gulf of California, supporting trans-Pacific dispersion. Our data on *S. lewini, C. falciformis* and *C. limbatus* supports this hypothesis as well. Comparing our NADH2 sequences to sequences from other regions in the world, reveals that ETP haplotypes were most similar to haplotypes from the Indo-Pacific Ocean, which implies that cross-Pacific dispersal has occurred in several shark species (Keeney & Heist, 2006).

Considering each species separately and understanding the status of populations at the species level is critical for the conservation management of exploited marine species (Sandoval-Castillo & Beheregaray, 2015), in order to establish suitable policies and conservation efforts that are regionally adapted to each of them. In this study, most species showed no population subdivision (with the exception of a single population of *M. lunulatus*), allowing for contemplating the studied Chocó region as a whole when developing management policies. However, collecting information of additional nuclear DNA markers is needed for more detailed evaluation of population structure and philopatry (bi-parental) on the species analyzed herein.

The high abundance in the area of endangered and CITES regulated species such as *S. lewini*, *C. falciformis* or *C. limbatus* (two of which are included in Appendix II of CITES), supports the importance of this region for shark conservation in the ETP. Populations of these species in the Colombian Tropical Pacific might be under an elevated risk of stock collapse due to their k-selected life history traits limiting the species recruitment abilities, their low genetic variability reducing their recovery potential from over-exploitation events and the increasing fishing pressure on their populations. Protective measurements such as reducing catch soaking time and release of live specimens have been mentioned by Feitosa et al. (2018) as powerful actions to reduce bycatch in Brazil contributing to the establishment of sustainable fisheries. We consider such approaches also adequate in the ETP and more effective compared to drastic fines. Additional measures should comprise a delimitation of conservation priority areas where fishing is banned at certain times of the year or certain fishing gear is not allowed to be used. Implemented at a regional level, such strategies could help to decrease shark bycatch in the Chocó area and improve the maintenance of shark populations in the Colombian Pacific and therefore in the Eastern Tropical Pacific.

## Conclusions

The coastal Chocó region previously represented a blank area on the map regarding chondrichthyan diversity in Colombia and its relation to the ETP with little previously published information about shark populations. Our study allows insights in shark species diversity landed as bycatch by fishermen, reflecting species occurrences in the waters of Chocó. Results presented herein are further useful for first characterization of populations, which is the basis for improving protective and management efforts in the region (Rodrigues-Filho et al., 2009). Based on our results we suggest the use of population genetics as a very useful and complementary tool to obtain first insights on the status of shark populations during the decision-making process when evaluating management and conservation strategies for shark species. Such information contributes to the improvement of fisheries regulations (Carreón-Zapiain et al., 2020), which can and should be replicated on a global scale. Our data exemplifies how different shark species face similar fishing pressures while in need of adapted conservation strategies depending on the intrinsic characteristics of the species. In addition, some of the species identified in this study have never been considered in the management plans for the Colombian Pacific waters before, which raises a great concern since most of them (all Sphyrnidae and Carcharhinidae members) are currently considered at high risk of stock collapse globally.

##  Supplemental Information

10.7717/peerj.13478/supp-1 Supplemental Information 1 Nucleotide composition per species based on mitochondrial NADH2 gene sequence data of sharks landed from bycatch along the northern Pacific Coast of Colombia

10.7717/peerj.13478/supp-2 Supplemental Information 2 Frequency of mitochondrial NADH2 haplotypes per species of sharks landed from bycatch along the northern Pacific Coast of Colombia

10.7717/peerj.13478/supp-3 Supplemental Information 3 Sequence Data

## Additional Information and Declarations

### Field Study Permissions

The following information was supplied relating to field study approvals (i.e., approving body and any reference numbers): Collection of samples in the field was approved by ANLA, Autoridad Nacional de Licencias Ambientales - Colombia (RCI0002-00-2021).

### Data Availability

The following information was supplied regarding data availability: The sequences are available at GenBank: OM165094 to OM165199.
