# Supplementary material for: Molecular identification and first demographic insights of sharks based on artisanal fisheries bycatch in the Pacific Coast of Colombia: implications for conservation
Source: PeerJ. 2022 Aug 4;10:e13478. doi: 10.7717/peerj.13478 (PMC9357375; doi:10.7717/peerj.13478)
Supplement: Supplemental Information 1 [file peerj-10-13478-s001.pdf]

Table S1: Nucleotide composition per species based on mitochondrial NADH2 gene sequence data of sharks landed from by-catch along the northern Pacific Coast of Colombia.

Species	C	T	A	G
Mustelus lunulatus	29.08%	30.01%	30.41%	10.49%
Sphyrna lewini	32.46%	27.12%	31.52%	8.91%
Mustelus henlei	9.97%	30.66%	29.72%	29.65%
Carcharhinus falciformis	29.22%	29.98%	31.71%	9.10%
Carcharhinus limbatus	8.82%	31.89%	29.40%	29.89%
