# Supplementary material for: Molecular identification and first demographic insights of sharks based on artisanal fisheries bycatch in the Pacific Coast of Colombia: implications for conservation
Source: PeerJ. 2022 Aug 4;10:e13478. doi: 10.7717/peerj.13478 (PMC9357375; doi:10.7717/peerj.13478)
Supplement: Supplemental Information 2 [file peerj-10-13478-s002.pdf]

Table S2: Frequency of mitochondrial NADH2 haplotypes per species of sharks landed from by-catch along the northern Pacific Coast of Colombia.

Haplotype	*Mustelus lunulatus*	*Sphyrna lewini*	*Mustelus henlei*	*Carcharhinus falciformis*	*Carcharhinus limbatus*
H1	58 (25.4%)	168 (79.2%)	12 (11.1%)	61 (84%)	20 (83.3%)
H2	39 (17.1%)	6 (2.83%)	9 (8.33%)	1 (1.39%)	2 (8.33%)
H3	3 (1.32%)	1 (0.47%)	3 (2.78%)	1 (1.39%)	2 (8.33%)
H4	38 (16.7%)	14 (6.6%)	10 (9.26%)	1 (1.39%)	-
H5	1 (0.43%)	5 (2.36%)	17 (15.7%)	1 (1.39%)	-
H6	17 (7.46%)	1 (0.47%)	6 (5.56%)	1 (1.39%)	-
H7	5 (2.19%)	1 (0.47%)	15(13.9%)	1 (1.39%)	-
H8	5 (2.19%)	1 (0.47%)	1 (0.92%)	3 (4.17%)	-
H9	2 (0.87%)	5 (2.36%)	1 (0.92%)	1 (1.39%)	-
H10	1 (0.43%)	1 (0.47%)	1 (0.92%)	1 (1.39%)	-
H11	2 (0.87%)	2 (0.94%)	7 (6.48%)	-	-
H12	6 (2.63%)	1 (0.47%)	8 (7.41%)	-	-
H13	2 (0.87%)	3 (1.42%)	4 (3.7%)	-	-
H14	2 (0.87%)	2 (0.94%)	2 (1.85%)	-	-
H15	5 (2.19%)	1 (0.47%)	5 (4.63%)	-	-
H16	1 (0.43%)	-	3 (2.78%)	-	-
H17	6 (2.63%)	-	2 (1.85%)	-	-
H18	1 (0.43%)	-	2 (1.85%)	-	-
H19	15 (6.58%)	-	-	-	-
H20	2 (0.87%)	-	-	-	-
H21	1 (0.43%)	-	-	-	-
H22	3 (1.32%)	-	-	-	-
H23	3 (1.32%)	-	-	-	-
H24	1 (0.43%)	-	-	-	-
H25	2 (0.87%)	-	-	-	-
H26	2 (0.87%)	-	-	-	-
H27	1 (0.43%)	-	-	-	-
H28	1 (0.43%)	-	-	-	-
H29	1 (0.43%)	-	-	-	-
H30	1 (0.43%)	-	-	-	-
H31	1 (0.43%)	-	-	-	-
